# Supplementary material for: Partitioning the roles of CYP6G1 and gut microbes in the metabolism of the insecticide imidacloprid in Drosophila melanogaster
Source: Sci Rep. 2017 Sep 12;7:11339. doi: 10.1038/s41598-017-09800-2 (PMC5595926; doi:10.1038/s41598-017-09800-2)
Supplement: Supplementary file 1 — Supplementary Information [file 41598_2017_9800_MOESM1_ESM.pdf]

Partitioning the roles of CYP6G1 and gut microbes in the metabolism of the insecticide imidacloprid in *Drosophila melanogaster*

Roberto Fusetto[1,2], Shane Denecke[2], Trent Perry[2], Richard A. J. O'Hair[1] and Philip Batterham[2]

[1]School of Chemistry, Bio21 Institute of Molecular Science and Biotechnology, University of Melbourne, Melbourne, Victoria, 3010, Australia.

[2]School of Bioscience, Bio21 Institute of Molecular Science and Biotechnology, University of Melbourne, Melbourne, Victoria, 3010, Australia.

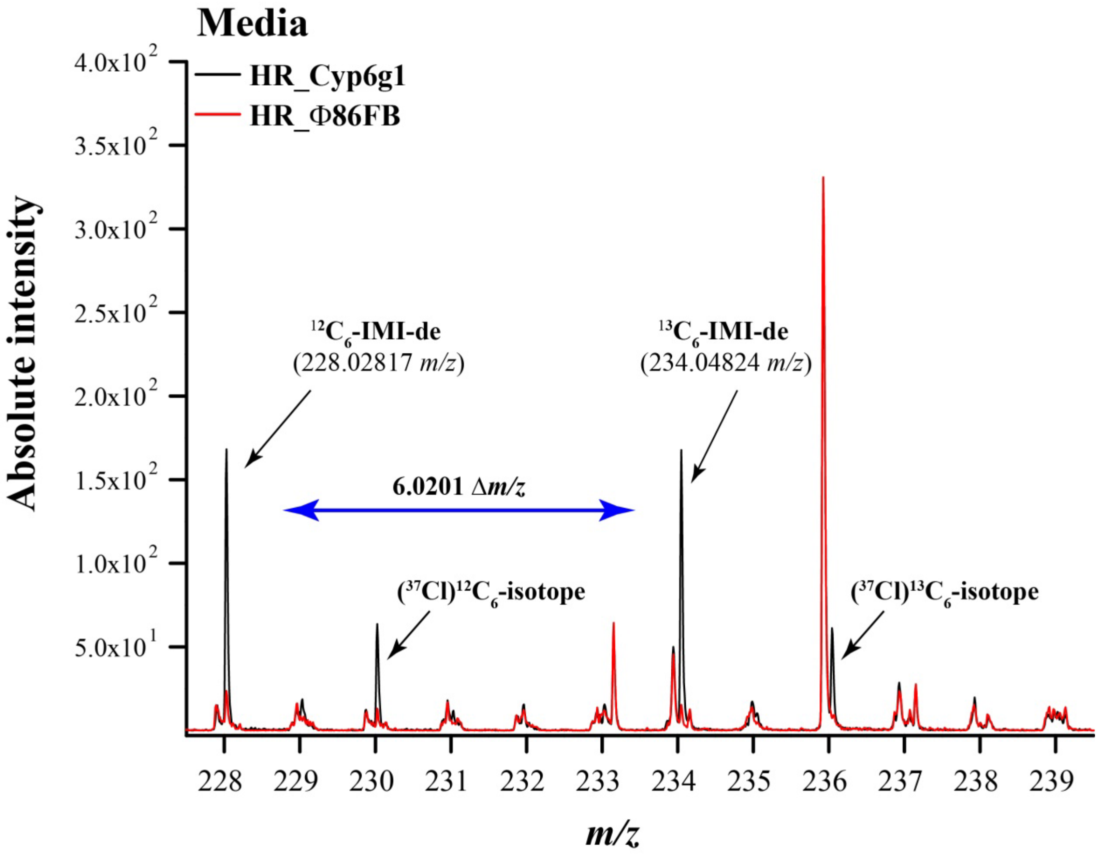

Supplementary Figure S1 The superimposed TIM mass spectrum of the HR_Cyp6g1 (black) and HR_Φ86FB (red) strains for IMI-de. The isotopic mass difference of 6.0201 mass units between the $^{12}C_6$ (228.02817 *m/z*) and the $^{13}C_6$- (234.04824 *m/z*) metabolite and the presence of the chlorine isotope (^{37}Cl) permitted IMI-de to be distinguished, even at low levels, from the various interferences present in the matrix. IMI-de was detected only in the media after 6 hours exposure to IMI.

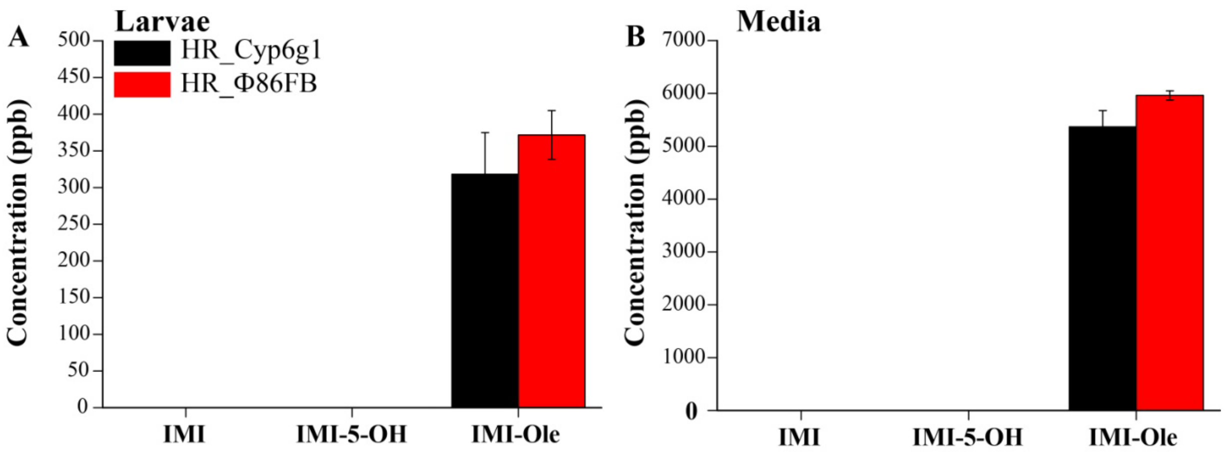

Supplementary Figure S2 The metabolism of IMI-Ole in the HR_Cyp6g1 (black) and HR_Φ86FB (red) strains. Lower, but not significant, levels of IMI-Ole are detected in both larval bodies (A) and exposure media (B) of the HR_Cyp6g1 strain. While metabolism of IMI-Ole might be explained through epoxidation on the imidazolidine ring[35], such a metabolite was not identified. IMI-Ole could not be reconverted into IMI-5OH, IMI or any of the other secondary metabolites generally observed in *D. melanogaster* [The data represent the mean±SD; (n=4); Student-t-test: P≤0.01 (**), P≤0.05 (*)].

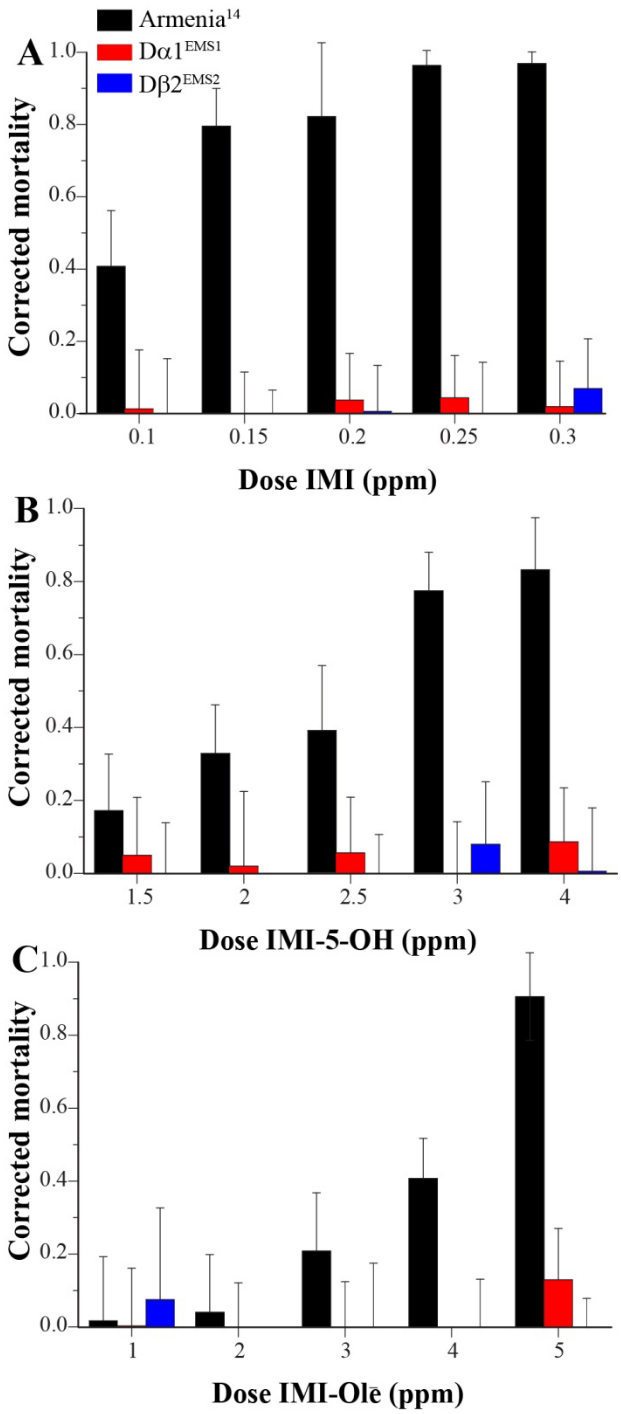

Supplementary Figure S3. Resistance to IMI (A), IMI-5-OH (B), and IMI-Ole (C) in *D.melanogaster*. The toxicity of IMI and major metabolites was measured using a larval bioassay on standard fly media as per Perry *et al* (2012)[60] (error bars - 95% Confidence intervals). The wildtype susceptible strain Armenia[14] and two EMS generated nicotinic acetylcholine receptor alleles, Dα1[EMS1], Dβ2[EMS2], previously reported as highly resistant to several neonicotinoids including IMI[61] were tested. Briefly, five replicates of 1st instar larvae (n=50) of the respective strains were placed on normal media or selected doses of IMI, IMI-5-OH or IMI-Ole in the dark at 25°C. Survival of fully enclosed adults was recorded at 16-18 days and mortality was corrected for control mortality using Abbott's formula[62] with confidence intervals calculated[63]. The results demonstrate that the three compounds are all toxic to wildtype larvae. The level of toxicity suggests that the order of toxicity in the assay is IMI>IMI-Ole>IMI-5-OH. IMI-5-OH is almost an order of magnitude lower in its effect on the survival of the larvae to adulthood. This could indicate that IMI-5-OH has a lower toxicity, it is not taken up from the media as readily or is quickly excreted from the larvae once ingested. The IMI resistant mutants are both resistant to the metabolites with no significant difference in the mortality to that of undosed media. These doses were sufficient to cause 80-90% mortality against the susceptible larvae.

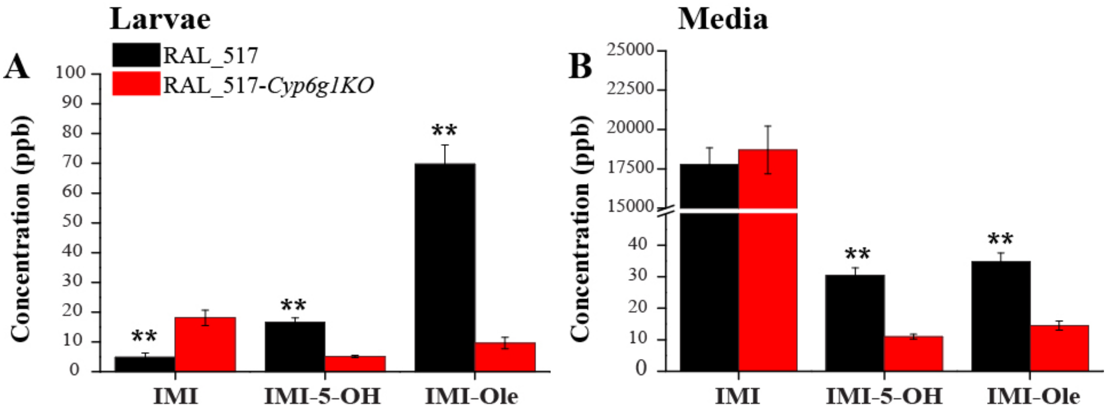

Supplementary Figure S4 IMI metabolism in the RAL_517-Cyp6g1KO at the same conditions reported by Denecke *et al* (this issue). IMI metabolism was monitored after 1 hr of exposure to a 25 ppm solution of IMI. The metabolism of IMI is significantly affected in the RAL_517-Cyp6g1KO mutant (red) compared to the RAL_517 control (black). As a consequence, significantly less IMI-5-OH and IMI-Ole was observed in the larval bodies (A) and media (B) of the RAL_517-Cyp6g1KO mutant compared to the RAL_517 control. Metabolites IMI-de and IMI-diol were observed at low levels only in the media of the RAL_517 strain (not reported in the graph) [The data represent the mean±SD; (n=4); Student-t-test: P≤0.01 (**), P≤0.05 (*)].

Rich media food	Grape juice
Soy flour (20 g)	Agar (20g)
Maize meal (73 g)	Yeast (7 g)
Yeast (35 g)	Sucrose (26 g)
Agar (6 g)	Dextrose (52 g)
Maltose (46 g)	Grape juice (200 mL)
Dextrose (75 g)	Tegosept (6 mL)
Acidic mix (14 mL)	Water (720 mL)
Tegosept (16 mL)	

Supplementary Table S1 Chemical composition rich media food and grape juice plates. The amount of each component is reported for a 1 L solution. The acid mix solution was made up of orthophosphoric acid (42 mL/L) and propionic acid (412 mL/L). The tegosept solution contained 50 g of methyl-p-hydroxy benzoate dissolved in 950 mL of 95% ethanol.

Supplementary References

60 Perry, T. *et al.* Effects of mutations in *Drosophila* nicotinic acetylcholine receptor subunits on sensitivity to insecticides targeting nicotinic acetylcholine receptors. *Pestic. Biochem. Physiol.* **102**, 56-60 (2012).

61 Perry, T., Heckel, D. G., McKenzie, J. A. & Batterham, P. Mutations in Dα1 or Dβ2 nicotinic acetylcholine receptor subunits can confer resistance to neonicotinoids in *Drosophila melanogaster*. *Insect Biochem. Mol. Biol.* **38**, 520-528 (2008).

62 Hoekstra, J. A. Acute bioassays with control mortality. *Water, Air, Soil Pollut.* **35**, 311-317 (1987).

63 Rosenheim, J. A. & Hoy, M. A. Confidence intervals for the Abbott's formula correction of bioassay data for control response. *J. Econ. Entomol.* **82**, 331-335 (1989).
